# miR-363 inhibits the growth, migration and invasion of hepatocellular carcinoma cells by regulating E2F3

**DOI:** 10.3892/or.2021.8181

**Published:** 2021-09-06

**Authors:** Junfeng Ye, Wei Zhang, Songyang Liu, Yahui Liu, Kai Liu

Oncol Rep 38: 3677-3684, 2017; DOI: 10.3892/or.2017.6018

Following the publication of this paper, it was drawn to the Editors’ attention by a concerned reader that tumour images featured in Fig. 2E strikingly similar to those that had already appeared in different form in another article by different authors at different research institutes. Owing to the fact that the contentious data in the above article had already been published elsewhere prior to its submission to *Oncology Reports*, the Editor has decided that this paper should be retracted from the Journal. After having been in contact with the authors, they agreed with the decision to retract the paper. The Editor apologizes to the readership for any inconvenience caused.

